# Pharmacokinetics of anti-infectious reagents in silkworms

**DOI:** 10.1038/s41598-019-46013-1

**Published:** 2019-07-01

**Authors:** Hiroshi Hamamoto, Ryo Horie, Kazuhisa Sekimizu

**Affiliations:** Teikyo University Institute of Medical Mycology, 359 Otsuka, Hachioji, Tokyo 192-0395 Japan

**Keywords:** Pharmacokinetics, Pharmacokinetics

## Abstract

Silkworm microorganism infection models are useful for screening novel therapeutically effective antimicrobial agents. In this study, we used silkworms to investigate the pharmacokinetics and metabolism of antimicrobial agents, in which cytochrome P450 plays a major role. The pharmacokinetic parameters of the antimicrobial agents were determined based on their concentrations in the hemolymph after administration. The parameters, such as half-lives and distribution volumes, in silkworm were consistent with those in mammalian models. In addition, antifungal agents with reduced therapeutic effectiveness due to high protein-binding capacities in mammalian serum exhibited similar features in silkworm hemolymph. Cytochrome P450 enzymes, which metabolize exogenous compounds in mammalian liver, were distributed mainly in the silkworm midgut. Most of the compounds metabolized by cytochrome P450 in humans are also metabolized in the silkworm midgut. These findings suggest that the pharmacokinetics of antimicrobial agents are fundamentally similar between silkworms and mammals, and that therapeutic effects in the silkworm infection model reflect the pharmacokinetics of the test samples.

## Introduction

Continued development of novel antimicrobial agents is crucial toward combatting the spread of multi-drug resistant microorganisms, which has become a serious problem worldwide. The discovery of novel antibiotics that are therapeutically effective and have distinct mechanisms from existing therapeutic options, however, is challenging. Mammals are generally used to evaluate the therapeutic effects of candidate compounds, but the associated high costs and animal welfare issues are prohibitive, prompting us to establish a silkworm larvae assay system to evaluate the therapeutic effectiveness of antimicrobials^1^. The half-maximal effective dose (ED_50_), i.e., the amount of the compound required to reduce survival of the microorganism by 50%, of clinically available antimicrobials is consistent between mammalian and silkworm models^2^. Using this silkworm whole body assay system, we recently obtained the novel antibiotics lysocin E^3^ and ASP2397^4^ by screening natural products, and a novel anti-methicillin resistant *Staphylococcus aureus* (MRSA) reagent, GPI0363, by screening a chemical library^5^. These compounds also had therapeutic effects in a murine systemic infection model. The mechanisms of metabolism are common between silkworms and mammals: cytochrome P450 activities are involved in phase I and conjugation enzymes are involved in phase II^6^. The precise pharmacokinetic parameters and protein-binding capacities of antimicrobials, which strongly affect their therapeutic effectiveness in silkworms, however, have not yet been determined. Where, what, and how exogenous chemicals are metabolized remain unanswered questions. In this report, we compared the pharmacokinetic parameters between silkworms and mammals, and found the values to be essentially similar. In addition, large amounts of cytochrome P450 enzymes were detected in the silkworm midgut, the organ responsible for metabolizing exogenous chemicals in the silkworm. Our results suggest that the therapeutic effectiveness of antimicrobials in the silkworm model reflect the pharmacokinetics of each compound, as in the case of mammalian models.

## Results

### Pharmacokinetic parameters of antimicrobials in silkworm

**Figure 1:**
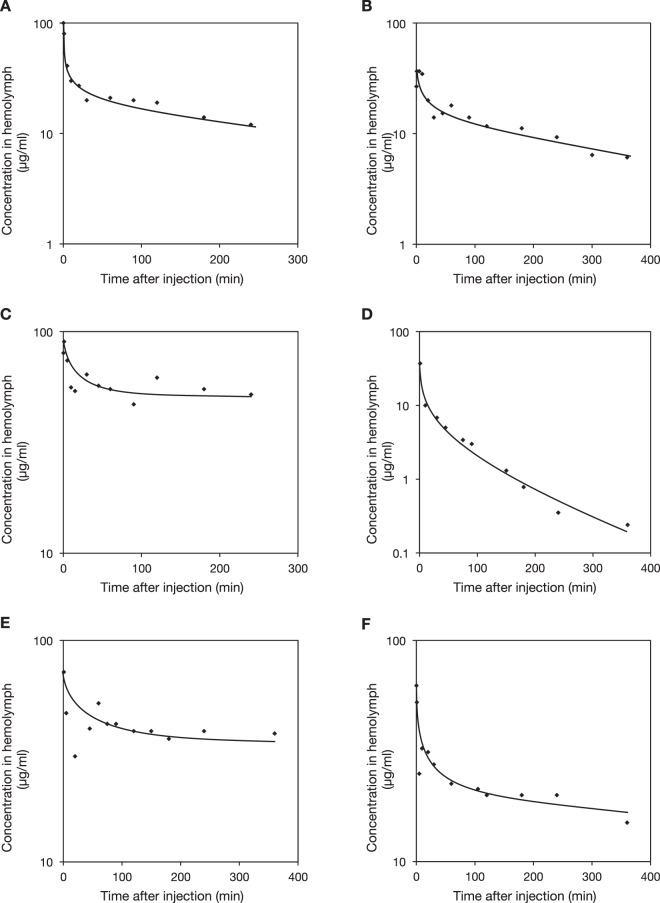
Time-course of the decrease in the concentration of antibiotics in silkworm hemolymph. Antibiotics were injected into silkworm hemolymph and hemolymph was collected by cutting the legs at the indicated time. Concentration of each antibiotic was determined by HPLC. (**A**) Chloramphenicol (100 µg/larva), (**B**) tetracycline (20 µg/larva), (**C**) vancomycin (50 µg/larva), (**D**) rifampicin (50 µg/larva), (**E**) micafungin (50 µg/larva), and (**F**) fluconazole (50 µg/larva).

We measured the pharmacokinetic parameters of antimicrobials, including half-lives, total clearance, and distribution volumes in the silkworm hemolymph based on the reaction rates of antimicrobials after their administration into the hemolymph. The time-courses of the decrease in the concentrations of antibacterial agents such as chloramphenicol, tetracycline, vancomycin, and rifampicin, and antifungal agents such as micafungin and fluconazole, are biphasic as in mammals: alpha phase, when the compounds rapidly distribute into the organs, and beta phase, when the compounds are eliminated by metabolism and excretion (Fig. 1, raw data are provided in the supplementary Dateset). They follow a two-compartment model. We applied this model to calculate the pharmacokinetic parameters in silkworms (Table 1). The distribution volumes (Vd_ss_) of the tested compounds were consistent between silkworms and mammals. In addition, differences in the total clearance and half-lives of the compounds were mostly within 2-fold; the maximum difference was 10-fold for the total clearance of rifampicin. The protein-binding capacities of antimicrobials affect their therapeutic efficacy due to decreased antimicrobial activity in a body fluid. We determined the protein-binding capacities and antifungal activities of ketoconazole and clotrimazole in silkworm hemolymph (Table 2). These reagents exhibit decreased antifungal activity in human serum due to their high protein-binding capacities. We demonstrated that they also exhibited high protein-binding capacities in silkworm hemolymph, which reduces their antifungal activities. Due to the attenuated antifungal activity by hemolymph proteins, much higher amounts of these antifungals were required to achieve therapeutic effectiveness. In contrast, fluconazole, which has a low protein-binding capacity in human serum, also had a low protein-binding capacity in silkworm hemolymph, and the reduction of antifungal activity is therefore attenuated. We found that fluconazole exhibited potent therapeutic effects at a low dose in the silkworm infection model (Table 2). Taken together, the pharmacokinetics parameters, such as half-lives, total clearance, distribution volumes, and protein-binding capacities, reflect the therapeutic effectiveness of antimicrobials in this silkworm infection model as in mammalian models.

**Table 1:** Pharmacokinetic parameters of antibiotics in silkworm and mammal.

Antibiotics	Silkworm				Mammal			
	Cl_tot_ (ml/g/h)	Vd_ss_ (ml/g)	t_1/2_ (h)	Dose (mg/kg)	Cl_tot_ (ml/g/h)	Vd_ss_ (ml/g)	t_1/2_ (h)	Dose (mg/kg)
Chloramphenicol	0.15	1.3	6.0	50	0.24	0.9	2.3	10
Tetracycline	0.07	0.6	5.5	10	0.35	1.0	2.0	10
Vancomycin	0.03	0.4	13.0	25	0.08	0.6	2.6	8
Rifampicin	2.11	3.1	1.0	25	0.31	1.3	2.9	10
Micafungin	0.03	0.5	12.0	25	0.07	0.4	5.0	1
Fluconazole	0.02	0.4	12.0	25	0.04	0.7	13.0	10

Total body clearance (Cl_tot_), distribution volume in steady state (Vd_ss_), and the elimination half-life (t_1/2_) was calculated based on time-course experiments (Fig. 1). Data for mammals (chloramphenicol^21^ and vancomycin^22^), rabbit (tetracycline^23^), sheep (rifampicine^24^), rat (micafungin^25^), and dog (fluconazole^26^) are cited from the indicated literature.

**Table 2:** Effect of protein binding on therapeutic activities of antifungal drug in silkworm.

Antibiotics	Protein binding (%)		MIC (µg/ml)		ratio (B/A)	ED_50_ (mg/kg)	ED_50_/MIC(A) ratio
	mammal	silkworm	without hemolymph (A)	with 10% hemolymph (B)			
Ketoconazole	99	>99	0.04	1.3	33	>10	>250
Clotrimazole	>98	>99	0.04	1.3	33	>10	>250
Fluconazole	10	65	0.4	3.2	8	0.7	1.8

The protein-binding capacity was determined using silkworm hemolymph as described in the Methods. The minimum inhibitory concentration (MIC) of each antifungal agent was determined in MHB with or without silkworm hemolymph. ED_50_ values of each antifungal agent in a silkworm infection model with *Candida albicans* were determined. Ratios between ED_50_ against MIC in the absence of hemolymph (MIC(A)) were calculated. Percentage of protein binding in human serum was cited from Schafer-Korting *et al*.^27^ for ketoconazole and fluconazole, and from Rosenkranzkw *et al*.^28^ for clotrimazole.

### Distribution of cytochrome P450 in silkworm organs

**Figure 2:**
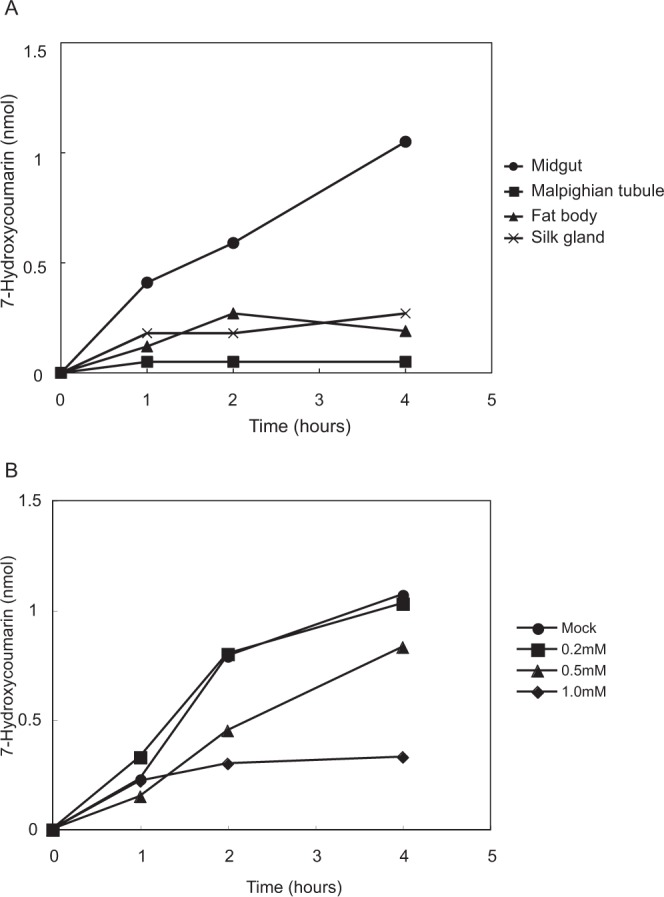
Tissue specificity of 7-ethoxycoumarin deethylation activity in silkworm. (**A**) The midgut, fat body, silk gland, and Malpighian tubule were extirpated from silkworms and incubated in medium containing 7-ethoxycoumarin at 30 °C. Medium was collected, extracted by acetone, and evaporated. Samples discovered in 50 mM NaOAc buffer (pH 6.0) were treated with beta-glucosidase. The amount of 7-hydroxycomarin, a metabolite, was determined by fluorometry. (**B**) Inhibition of 7-ethoxycoumarin deethylation by cimetidine in the silkworm midgut. Silkworm midgut was incubated in medium containing 7-ethoxycoumarin and cimetidine at 30 °C. Samples were collected, extracted by acetone, and treated with beta-glucosidase. The amount of 7-hydroxycomarin was determined by fluorometry.

In mammals, including humans, metabolic reactions by cytochrome P450 in the liver play a crucial role in the pharmacokinetics of exogenous compounds such as antimicrobials. Many organisms have their own cytochrome P450 species and metabolize exogenous compounds. For example, rifampicin and azoles are metabolized by CYP450 3A4 in the human liver. In silkworms, at least 79 genes encoding cytochrome P450 have been identified by whole genome sequencing^7^. In silkworms, cytochrome P450 is present in the ecdysone synthetic pathway^8^ and is necessary for the detoxification of insecticides^9^, but information regarding the function of P450 for the metabolism of antibiotics and other exogenous chemicals is limited. We previously reported that 7-ethoxycoumarine, a model compound metabolized by CYP450 2B6 in mice^6^, is also metabolized in silkworms. In the present study, we attempted to identify the organs responsible for metabolism of the compound by cytochrome P450 enzymes using an *in vitro* organ culture^2^. We found that the midgut, not the fat body, had the highest capacity to metabolize 7-ethoxycoumarin, and cimetidine, a CYP450 inhibitor, inhibited this reaction in the midgut (Fig. 2). In addition, the midgut contains 67% of the cytochrome P450 enzymes based on analysis of the CO-difference spectra of whole organs (Table 3). These results suggest that the midgut is responsible for metabolizing exogenous chemicals via cytochrome 450 enzymes.

**Table 3:** Amounts of cytochrome P450 in silkworm tissue.

Tissue	Amount of Cytochrome P450		
	(pmol/mg microsomal protein)	pmol/total tissue	Ratio (%)
Midgut	30.7	460	67
Fat body	<1.8	<10	
Malpighian tubule	<10.0	<10	
Silk gland	<6.3	<10	
Head	26.3	100	17
Skin and Muscle	17.6	120	15

Organs were extirpated from silkworms and microsome fractions were prepared. The amounts of cytochrome P450 were measured by CO difference spectra.

### Biochemical analyses of cytochrome P450 in the silkworm midgut

**Figure 3:**
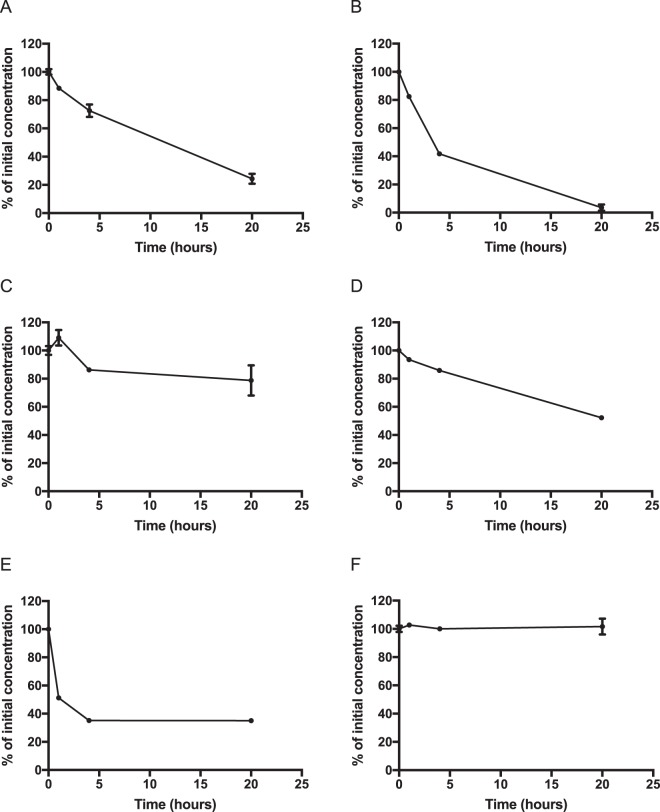
Decay of cytochrome P450 substrates in *in vitro* culture of the silkworm midgut. Silkworm midgut was incubated in medium with cytochrome P450 substrates at 30 °C. The samples were collected, extracted by acetone, and evaporated. The amounts of substrates were determined by HPLC analysis. (**A**) Omeprazole, (**B**) rifampicin, (**C**) ketoconazole, (**D**) diclofenac, (**E**) testosterone, and (**F**) caffeine.

We further tested whether a variety of compounds metabolized by cytochrome P450 in mammalian liver are also metabolized in the silkworm midgut. In humans, ketoconazole, rifampicin, omeprazole, diclofenac, and testosterone are metabolized by CYP 3A4, 3A4, 2C19, 2C9, and 3A5, respectively. These compounds were metabolized in the silkworm midgut in an *in vitro* organ culture (Fig. 3A–E, raw data are provided in the supplementary Dataset). On the other hand, caffeine, metabolized by CYP 1A2 in humans, was not metabolized in the silkworm midgut, even after 20 h of incubation (Fig. 3F). Therefore, some compounds like caffeine cannot be metabolized by the silkworm. In addition, we examined a group of luciferin derivatives metabolized by specific cytochrome P450 enzymes in the human liver (Table 4). In silkworms, all tested luciferin derivatives were metabolized in the midgut (Table 4). These findings suggest that the silkworm midgut is able to metabolize various compounds that are metabolized by cytochrome P450 in the human liver.

**Table 4:** Metabolism of luciferin derivative in the silkworm midgut.

Substrate	Cytochrome P450 in human	RFU/organ
Luciferin-ME	1A2, 2C8, 2C9, 2J2, 4A11, 4F3B, 19	58,500
Luciferin-CEE	1A1, 1B1, 3A7	17,900
Luciferin-PFBE	3A4, 3A5, 3A7	229,000
Luciferin-IPA	3A4	198,000

Silkworm midgut was incubated in medium containing luciferin substrates at 30 °C. Production of luciferin was determined by a luminometer.

Cytochrome P450 enzymes are present in microsomal fractions prepared from mammalian livers. We prepared microsomal fractions from the silkworm midgut and examined the metabolism of luciferin-ME. The Km value determined by a Lineweaver-Burk plot was 7.8 µM and Vmax was 3.0 pmol/min/mg protein, consistent with the values of other luciferin compounds obtained from microsomal fractions of human liver (Supplementary Fig. 1)^10^. These findings suggest that microsomal fractions from the silkworm midgut have metabolism capacities corresponding to those of mammals.

## Discussion

In this report, we examined the pharmacokinetics of exogenous compounds in silkworm. We determined the pharmacokinetic parameters, such as half-lives, distribution volumes, and total clearance, by measuring the rate of decrease of drug concentrations in the hemolymph. We previously demonstrated that ED_50_ values of various antibiotics are consistent between silkworm and mouse models^2^, suggesting that the pharmacokinetic parameters of antibiotics in the silkworm are similar to those in mammals. Our present results showed that the pharmacokinetic parameters of antibiotics in the silkworm are also similar to those in mammalian models. In addition, the time-course of the decrease in drug concentrations in the hemolymph is explained by a two-component model, as in mammalian models. Furthermore, the protein-binding capacities of antifungal agents, which critically affect their therapeutic efficacies, were consistent between silkworm and mammalian models. These results suggest that the therapeutic effectiveness of antibiotics in a silkworm model reflects the pharmacokinetics of the compounds, as in mammalian models. Insects such as the silkworm have an open vessel system, whereas mammals have a closed vessel system. Although the circulatory systems are very different between silkworms and mammals, the silkworm has a dorsal vessel to mix the hemolymph, similar to the mammalian heart, which circulates blood throughout the whole body in a minute. We consider that compounds injected into the silkworm hemolymph distribute rapidly into the whole body as in mammalian models.

In addition to the silkworm, wax moth^11–13^ and grasshopper^14^ are insect animal models reported to be available for evaluating the therapeutic effectiveness of antimicrobials. Some reports determined the C_max_, half-life, and area under the curve values by measuring drug concentrations in the insect hemolymph. In these reports, the half-lives of cefditoren and imipenem were consistent with those in a mammalian model, but the pharmacokinetic compartment was not identified. Together with our results obtained in silkworm, these findings suggest that fundamental mechanisms involved in the pharmacokinetics of antibiotics are common between insects and mammals.

It has remained unclear in insects whether cytochrome P450 enzymes contribute to the metabolism of exogenous drugs, because little attention has been paid to insects as alternative model animals for drug development. In this report, we examined the functions of cytochrome P450 enzymes in the silkworm intestine. We revealed that compounds metabolized by cytochrome P450 in the human liver were also metabolized in the silkworm midgut. Most of the known cytochrome P450 enzymes are present in the silkworm midgut. In mammals, exogenous chemicals are absorbed from the intestine and transferred to the liver via the portal vein, and then metabolized by P450 enzymes. Thus, toxic compounds or drugs are inactivated in the liver before distribution into the whole body, a process called the “first pass effect”. In silkworms, exogenous compounds absorbed from the intestine are directly transferred to the hemolymph, immediately exposing all the tissues and organs to the compounds. Therefore, it is reasonable that most exogenous compounds are metabolized by the silkworm midgut before distribution throughout the whole body. Actually, several types of cytochrome P450 are highly expressed in the silkworm midgut^7^, and oral administration of phoxim, an insecticidal compound, induces the expression of cytochrome P450 genes in the midgut^15^. In addition, the amount of cytochrome P450 and induction of cytochrome P450 in the midgut by feeding of exogenous compounds is higher than that in the fat body in larvae of the southern armyworm^16^ and cotton bollworm^17^. Thus, this situation seems to be common to all invertebrate animals with open vessel systems. It is important to note, however, that the fat body, which is known to metabolize exogenous compounds, may contribute to metabolize exogenous compounds not completely metabolized in the midgut. In fact, cytochrome P450 in the fat body is also increased after exposure to exogenous compounds^16,17^. Regardless of the involvement of cytochrome P450 in silkworm metabolism, we could not analyze metabolites of the exogenous compounds. Future studies should investigate the similarities of the metabolic reactions by cytochrome P450 in silkworms compared with mammals.

In conclusion, the findings of the present study demonstrate that silkworms are reliable alternative model animals for identifying therapeutically effective antimicrobial agents. Only a limited number of compounds, mainly antibiotics, were tested in this study. To establish the silkworm system as an experimental model for screening drug candidates, further studies of the pharmacokinetics of various medicines, including antibiotics, are necessary.

## Materials and Methods

### Animals and reagents

Silkworms, *Bombyx mori* (Hu•Yo × Tsukuba•Ne) were reared from eggs (Ehime Sanshu, Ehime, Japan) using artificial food (Silkmate 2S, Katakura Industries Co., Ltd. Tokyo, Japan) until the 5th instar^2^. The reagents and antibiotics used in these experiments were purchased from MilliporeSigma (St. Louis, MO) or Fujifilm Wako Pure Chemical (Tokyo, Japan).

### Measurement of pharmacokinetics of antibiotics in the silkworm model

Antibiotics dissolved in saline were injected into the hemolymph of 5th instar silkworms using a 1-mL syringe attached to a 27 G needle. Hemolymph was harvested from abdominal appendages by cutting them with scissors at the indicated time-points after administration, and mixed with an equal volume of acetone. Samples were centrifuged at 15,000 rpm for 5 min at 4 °C, and the supernatants were evaporated with a centrifuge evaporator^18^. The dried materials were dissolved in 50% methanol and filtrated through a 0.45-µm filter. The samples were analyzed by a high-pressure liquid chromatography (HPLC, pump model 600E and PDA detector 2996, Waters, Milford, MA, with PEGASIL ODS SP100 [φ4.5 mm × 250 mm, Senshu Scientific, Tokyo, Japan] at 1 mL/min, isocratic mode). The amount of antibiotics in the sample was quantified from the peak area of the chromatogram. The mobile phase used comprised acetonitrile:20 mM Na_2_HPO_4_ = 92:8 for vancomycin, methanol:5 mM EDTA = 30:70 for tetracycline, acetonitrile:20 mM potassium phosphate buffer = 45:55 for chloramphenicol, 40% acetonitrile with 0.05% formic acid for rifampicin, 40% acetonitrile with 0.1% trifluoroacetic acid for micafungin, acetonitrile:25 mM acetic acid buffer (pH 5.0) = 1:4 for fluconazole, 30% acetonitrile with 0.05% formic acid for ketoconazole, and 40% acetonitrile (pH was adjusted to 3 with phosphoric acid) for clotrimazole.

### Measurement of antimicrobial and antifungal activities

Antimicrobial and antifungal activities of each compound were measured by a micro dilution method^19,20^. To measure protein binding to antifungal activity, silkworm hemolymph was collected by cutting the pseudopodium using scissors, and centrifuged at 3000 rpm for 5 min at 4 °C. Phenylthiourea (final concentration 1 mM) and gentamicin (final concentration 50 µg/mL) were added to the supernatant, and the samples were used for assay after 2-fold serial dilution with RPMI 1640 medium.

### Measurement of protein-binding capacities of antifungal antibiotics

Each antifungal antibiotic was dissolved into silkworm hemolymph collected as described above, at 10 µM and incubated at 27 °C for 10 min. After centrifugation at 14,000 × g with a filter unit (3000 MW, Millipore, Bedford, MA), the filtrate was mixed with the same volume of methanol to remove proteins and centrifuged, and the supernatant was analyzed by HPLC.

### Metabolism of compounds in silkworm cultured tissues

Silkworms (5th instar, 5 days) were dissected in a buffer (10 mM HEPES/NaOH, 0.9% NaCl, pH 7.9), and isolated organs were incubated with 3 mL TC-100 medium containing 10% fetal bovine serum, 50 µg/ml gentamicin, and substrates for the metabolic reaction. Whole samples (medium and organ) were homogenized and extracted by equal volumes of acetone followed by evaporation. Extracted samples were dissolved in 50 mM NaOAc buffer and treated with beta-glucosidase to measure the fluorescence of metabolized 7-ethoxycoumarin^6^. Fluorescence was measured (Em: 460 nm, Ex: 380 nm) for each 100-µL sample in 3 mL of glycine buffer by fluorometry (Jasco, FP-1000, Tokyo, Japan). Samples for HPLC were dissolved in 50% methanol. HPLC analysis was performed using a PEGASIL ODS SP column (φ4.5 mm × 250 mm) with isocratic elution at a flow rate of 1 ml/min. For luciferin derivatives, incubated samples in silkworm cultured tissues were mixed with detection reagent (P450-Glo Assay, Promega, Madison, WI), and luminescence was measured by a luminometer (Lumat LB 9507, Berthold Technologies, Bad Wildbad, Germany).

### Preparation of microsome fraction

Silkworms were immersed in buffer (10 mM HEPES/NaOH, 0.9% NaCl, pH7.9), and organs were isolated and homogenized in a buffer (100 mM sodium phosphate, 1 mM EDTA, 2 mM benzamidine, 10% glycerol) by a glass-Teflon homogenizer. The tissue homogenates were centrifuged at 9000 × g for 10 min at 4 °C. The supernatant was further centrifuged at 49,000 rpm for 30 min at 4 °C. The precipitate was suspended in buffer (100 mM sodium phosphate, 1 mM EDTA, 2 mM benzamidine, 10% glycerol) and frozen at −80 °C after flash-freezing with liquid nitrogen until use.

### Carbon monoxide difference spectra analysis

The microsome fractions were reduced with a few milligrams of Na_2_S_2_O_4_ and the spectra were measured. Samples were treated with CO and the difference spectra were measured using a Shimazu UV-3000.

### Cytochrome P450 reaction *in vitro*

Microsomes and substrates were suspended in final 0.25 ml buffer comprising 0.1 M sodium phosphate buffer (pH 7.5) and 0.1 mM EDTA, and pre-incubated for 5 min at 30 °C. A cytochrome P450 reaction was initiated by adding a NADPH-generating system containing 3.3 mM glucose-6-phosphate, 1.25 mM NADP, 3.3 mM MgCl_2_, and 0.25 U glucose-6-phosphate dehydrogenase as a final concentration.

## Supplementary information

Supplementary Information

Supplementary Dataset

## References

[1] Kaito C, Akimitsu N, Watanabe H, Sekimizu K (2002). Silkworm larvae as an animal model of bacterial infection pathogenic to humans. Microb Pathog.

[2] Hamamoto H (2004). Quantitative evaluation of the therapeutic effects of antibiotics using silkworms infected with human pathogenic microorganisms. Antimicrob Agents Chemother.

[3] Hamamoto H (2015). Lysocin E is a new antibiotic that targets menaquinone in the bacterial membrane. Nat Chem Biol.

[4] Nakamura I (2017). Discovery of a new antifungal agent ASP2397 using a silkworm model of *Aspergillus fumigatus* infection. J Antibiot (Tokyo).

[5] Paudel A (2017). A Novel Spiro-Heterocyclic Compound Identified by the Silkworm Infection Model Inhibits Transcription in *Staphylococcus aureus*. Front Microbiol.

[6] Hamamoto H, Tonoike A, Narushima K, Horie R, Sekimizu K (2009). Silkworm as a model animal to evaluate drug candidate toxicity and metabolism. Comp Biochem Physiol C Toxicol Pharmacol.

[7] Li B (2014). Identification and characterization of six cytochrome P450 genes belonging to CYP4 and CYP6 gene families in the silkworm, *Bombyx mori*. Mol Biol Rep.

[8] Niwa R (2004). CYP306A1, a cytochrome P450 enzyme, is essential for ecdysteroid biosynthesis in the prothoracic glands of *Bombyx* and *Drosophila*. J Biol Chem.

[9] Li F (2015). Expression profile analysis of silkworm P450 family genes after phoxim induction. Pestic Biochem Physiol.

[10] Li AP (2009). Evaluation of luciferin-isopropyl acetal as a CYP3A4 substrate for human hepatocytes: effects of organic solvents, cytochrome P450 (P450) inhibitors, and P450 inducers. Drug Metab Dispos.

[11] Chibebe Junior J (2013). Photodynamic and antibiotic therapy impair the pathogenesis of *Enterococcus faecium* in a whole animal insect model. PLoS One.

[12] Desbois AP, Coote PJ (2011). Wax moth larva (*Galleria mellonella*): an *in vivo* model for assessing the efficacy of antistaphylococcal agents. J Antimicrob Chemother.

[13] Peleg AY (2009). *Galleria mellonella* as a model system to study Acinetobacter baumannii pathogenesis and therapeutics. Antimicrob Agents Chemother.

[14] Siddiqui R, Osman K, Khan NA (2012). A novel *in vivo* model to study bacterial pathogenesis and screen potential therapeutic targets. J Med Microbiol.

[15] Gu Z (2014). The adverse effects of phoxim exposure in the midgut of silkworm, *Bombyx mori*. Chemosphere.

[16] Brattsten LB, Price SL, Gunderson CA (1980). Microsomal oxidases in midgut and fatbody tissues of a broadly herbivorous insect larva, *Spodoptera eridania* cramer (Noctuidae). *Comparative Biochemistry and Physiology Part C: Comparative*. Pharmacology.

[17] Qiu X, Li W, Tian Y, Leng X (2003). Cytochrome P450 monooxygenases in the cotton bollworm (Lepidoptera: Noctuidae): tissue difference and induction. J Econ Entomol.

[18] Fujiyuki T, Imamura K, Hamamoto H, Sekimizu K (2010). Evaluation of therapeutic effects and pharmacokinetics of antibacterial chromogenic agents in a silkworm model of *Staphylococcus aureus*. infection. Drug Discov Ther.

[19] Clinical and Laboratory Standards Institute. *Methods for dilution antimicrobial susceptibility tests for bacteria that grow aerobically; approved standard—Eighth edition CLSI document M07-A8*. Vol. 29 (Clinical and Laboratory Standards Institute, 2009).

[20] Clinical and Laboratory Standards Institute. *Reference method for broth dilution antifungal susceptibility testing of yeasts: Approved Standard M27-A*. (Clinical and Laboratory Standards Institute, 1997).

[21] Koup JR, Lau AH, Brodsky B, Slaughter RL (1979). Chloramphenicol pharmacokinetics in hospitalized patients. Antimicrob Agents Chemother.

[22] Aldaz A, Ortega A, Idoate A, Giraldez J, Brugarolas A (2000). Effects of hepatic function on vancomycin pharmacokinetics in patients with cancer. Ther Drug Monit.

[23] Percy DH, Black WD (1988). Pharmacokinetics of tetracycline in the domestic rabbit following intravenous or oral administration. Can J Vet Res.

[24] Sweeney RW, Divers TJ, Benson C, Collatos C (1988). Pharmacokinetics of rifampin in calves and adult sheep. J Vet Pharmacol Ther.

[25] Niwa T (2004). Tissue distribution after intravenous dosing of micafungin, an antifungal drug, to rats. Biol Pharm Bull.

[26] Humphrey MJ, Jevons S, Tarbit MH (1985). Pharmacokinetic evaluation of UK-49,858, a metabolically stable triazole antifungal drug, in animals and humans. Antimicrob Agents Chemother.

[27] Schafer-Korting M, Korting HC, Amann F, Peuser R, Lukacs A (1991). Influence of albumin on itraconazole and ketoconazole antifungal activity: results of a dynamic *in vitro* study. Antimicrob Agents Chemother.

[28] Rosenkranzkw H, PÜtter J (1976). The binding of Clotrimazole to the proteins of human serum. Eur J Drug Metab Pharmacokinet.

