# Supplementary material for: Pharmacokinetics of anti-infectious reagents in silkworms
Source: Sci Rep. 2019 Jul 1;9:9451. doi: 10.1038/s41598-019-46013-1 (PMC6602958; doi:10.1038/s41598-019-46013-1)
Supplement: Supplementary file 1 — Supplementary Information [file 41598_2019_46013_MOESM1_ESM.docx]

Supplementary Information for

**Pharmacokinetics of anti-infectious reagents in silkworms**

Hiroshi Hamamoto, Ryo Horie, Kazuhisa Sekimizu

Teikyo University Institute of Medical Mycology, 359 Otsuka, Hachioji, Tokyo, 192-0395, Japan

**Supplementary Figure 1 Metabolism of luciferin-ME by cytochrome P450 in an *in vitro* assay system**

Microsome fractions were prepared from the silkworm midgut, and incubated with luciferin-ME for 5 min at 30°C. The reactions were initiated by the addition of an NADPH-regeneration system. Production of luciferin was determined using a luminometer. (a) Titration of luciferin-ME. (b) Lineweaver Burk plot made from the titration curve.
